# Associations between professional values and self-actualization among South Korean and Chinese medical students

**DOI:** 10.5116/ijme.6ac6.3280

**Published:** 2026-10-15

**Authors:** Hyojeong Hwang, Jun-Ki Lee, Qiuyue Fang, Hyo Hyun Yoo

**Affiliations:** 1 Institute of Science Education, Jeonbuk National University, Jeonju, Republic of Korea; 2 Division of Science Education, Biology Major, Institute of Science Education, Jeonbuk National University, Jeonju, Republic of Korea; 3 The Wilson Centre, University Health Network, Toronto, Ontario, Canada; 4 Department of Medical Education, College of Medicine, Jeonbuk National University, Jeonju, Republic of Korea

**Keywords:** Professionalism, students, medical, cross-cultural comparison, self-actualization, motivation

## Abstract

**Objectives:**

To compare professional values endorsed at one South Korean and one Chinese medical school, examine which values were associated with endorsement of self-actualization, and identify latent value classes.

**Methods:**

Cross-sectional convenience survey of 321 medical students: 211 second- and third-year students at a Korean national university, 110 at a Chinese medical college. Twelve items (11 values, self-actualization) were scored as endorsed or not, with unlimited selections; the Chinese version was back-translated. Analyses were t-tests, phi coefficients, logistic regression, and latent class analysis with Benjamini–Hochberg correction.

**Results:**

Competence was endorsed most frequently in both samples. Korean students endorsed competence, t_(319)_ = 3.27, p = .001, and collaboration, t_(319)_ = 4.05, p < .001, more often, Chinese students endorsed social recognition, t_(319)_ = −3.01, p = .003; all survived correction, with small effects (d = 0.38, 0.48, −0.35). Satisfaction with the major was higher in the Korean sample, t_(318) _= 27.66, p < .001, d = 3.26, reported descriptively only. Social contribution was associated with self-actualization in both samples (r = .25 Korean, .44 Chinese) and was the only logistic coefficient to remain significant after adjustment (OR = 5.42, p = .001, Chinese sample); Korean associations attenuated after adjusting for the number of values endorsed. Latent classes (two Korean, three Chinese) differed mainly in endorsement breadth.

**Conclusions:**

Social contribution was the most consistent correlate of endorsement of self-actualization. Because each country was represented by one institution, differences cannot be attributed to culture. Career guidance may address value heterogeneity within cohorts; multi-institutional longitudinal research is needed.

## Introduction

Professional values serve as internal criteria that reflect an individual's goals and beliefs about their profession. These values are associated with career choices, professional commitment, and job satisfaction.^1^^,^^2^ They encompass desirable attitudes toward one's occupation and the values pursued, making them psychological factors linked to overall life satisfaction.^3^^,^^4^ Professional values play an especially critical role in fields that require high responsibility and ethical standards, such as medicine, serving as a moral foundation for the development of professional identity.^5^^-^^8^

For medical students, professional values are central to shaping their future identities as healthcare professionals.^6^^-^^8^ Given that their work involves handling life and health, they must internalize high ethical standards and a strong sense of social responsibility, which is associated with professional commitment.^5^^,^^7^^,^^9^ Throughout medical education, establishing an understanding of ethical dilemmas alongside professional identity is essential for students' development into competent medical professionals.^6^^-^^8^

Previous research has primarily focused on how professional values relate to career choice, job satisfaction, and achievement, with an emphasis on individual aspects.^1^^,^^10^ However, other studies highlight the role of social structures and educational contexts in the formation of professional values, which vary across settings.^2^^,^^11^^-^^14^ These findings suggest professional values are not merely personal preferences but are also socially situated constructs that reflect institutional and societal expectations. Studies among medical students similarly report that contextual factors are associated with the formation of professional values.^14^^-^^16^

Self-Determination Theory (SDT) describes how individuals internalize social norms as personal values by satisfying the needs for autonomy, competence, and relatedness.^17^ SDT is used in the present study as an interpretive framework only; none of its constructs were directly measured, and the study therefore cannot test SDT propositions. Hofstede's framework of cultural dimensions describes how orientations such as individualism and collectivism are associated with motivational priorities across societies,12 and is likewise used here only as a descriptive lens for discussing findings, not as a measured variable.

As international collaboration in medicine increases, academic and educational exchanges in medical education have also expanded.^18^^-^^21^ This underscores the need to prepare future medical professionals to understand and work within multicultural contexts.^22^^-^^26^ Describing variation in professional values across educational settings is useful for recognizing differences in medical curricula, societal expectations of physicians, and the profession's standing. Comparative descriptive studies of medical students' professional values in different countries nevertheless remain limited, and most existing work is confined to single settings.

The present study, therefore, compared the professional values endorsed by medical students at one Korean and one Chinese institution and examined how these values were associated with endorsement of self-actualization. Because the two samples were drawn from single institutions, the comparison is presented as a comparison between two student groups rather than between two national cultures; institutional, curricular, and admissions differences cannot be separated from any broader contextual differences.14 Recognizing this constraint, the study was framed as exploratory and descriptive rather than as a test of cultural hypotheses. Self-actualization was operationalized as a single binary indicator: students either did or did not select "self-actualization" from the same 12-item list used for the other value items, following the source inventory's design.^27^ This single-item, binary operationalization means that the study can speak only to whether students endorsed self-actualization as an important professional value, and not to any level, degree, or developmental stage of self-actualization.

Guided by these considerations, three research questions were addressed. First, how did the endorsement of professional values and satisfaction with major differ between the two student groups? Second, which professional values were associated with endorsement of self-actualization in each group? Third, what latent value classes could be identified within each group, and did endorsement of self-actualization differ across these classes?

## Methods

### Study design and participants

This study used a cross-sectional, questionnaire-based design. The participants were 321 medical students: 211 second- and third-year students at a national university in the southern region of South Korea and 110 students at a medical college in Shandong Province, China, who were at the training stage corresponding to the second year of the Korean medical course. Because the two curricula are organized differently, this correspondence is approximate. Participants were recruited via convenience sampling: all students available during the single data-collection period were invited to participate, and no sample-size calculation was performed. Students who did not consent or who returned incomplete questionnaires were excluded. Participant characteristics are reported in Table 1.

The two samples were similar in sex composition but differed in year composition (Table 1). Because the groups were not matched on these characteristics, and because each country was represented by a single institution, all between-group comparisons are descriptive contrasts that may partly reflect differences in the composition of the two samples rather than differences attributable to country.

The study protocol was approved by the Institutional Review Board of Jeonbuk National University, and the responsible authorities at the participating Chinese institution granted permission for data collection; all procedures conformed to the Declaration of Helsinki. Participation was voluntary and anonymous, written informed consent was obtained before completion of the questionnaire, and students were told that participation or non-participation would not affect their grades or academic standing.

### Instrument

This study assessed professional values using the 11 value categories of the work-values inventory developed by Lim and colleagues at the Korea Research Institute for Vocational Education and Training.^27^ The categories are competence, diversity, remuneration, stability, social recognition, leadership, collaboration, social contribution, career development, creativity, and autonomy. Self-actualization was presented as a twelfth item in the same list. Participants were asked to select every item they considered an important professional value; the questionnaire imposed no limit on the number of items they could select.

**Table 1:** Characteristics of the participants

Characteristic	Korean sample (n = 211)	Chinese sample(n = 110)	Test statistic	p
Female, n (%)	93 (44.1)	58 (52.7)	χ²(1) = 2.17	.141
Male, n (%)	118 (55.9)	52 (47.3)	Cramér's V = .08	
Year 2 or equivalent, n (%)	118 (55.9)	110 (100.0)	χ²(1) = 68.26	< .001
Year 3, n (%)	93 (44.1)	0 (0.0)	Cramér's V = .46	
Satisfaction with major, M (SD)	4.36 (0.77)	1.89 (0.73)	t_(__318)_ = 27.66	< .001
Number of the 11 values endorsed, M (SD)	4.15 (2.48)	3.89 (2.51)	t_(__319)_ = 0.87	.383
Number of the 11 values endorsed, range	0–11	0–11		

Note. Percentages are within-sample column percentages. All Chinese participants were at the training stage corresponding to the second year of the Korean medical course; because the two curricula are organized differently, this correspondence is approximate. Satisfaction with the major was rated on a five-point scale (1 = not at all satisfied, 5 = very satisfied); in both language versions, the printed options ran from the most to the least favorable response and were therefore reverse-coded before analysis. The between-group difference on this single item is very large and is reported only descriptively. One Korean participant did not answer this item. Age was not collected.

Each of the 12 items was therefore scored dichotomously as selected (1) or not selected (0), and the same items, instructions, and coding scheme were used in both institutions.

The instrument was independently translated into Chinese and back-translated into Korean by two bilingual specialists in medical education, a procedure that establishes only semantic correspondence. The inventory was originally developed for Korean secondary and vocational education; no psychometric evaluation of its performance among Chinese medical students has been reported, and measurement invariance was not tested, so it cannot be assumed that the items carry equivalent meaning in the two samples.^28^ For the same reason, no claim of established reliability is made; internal-consistency indices are not meaningful for a checklist of conceptually distinct, independently selected items.

Participants also reported their sex, academic year, and satisfaction with their major, the last on a single five-point Likert-type item. In both language versions, the response options were printed from the most to the least favorable category, so responses in both samples were reverse-coded before analysis, with higher values denoting greater satisfaction (1 = not at all satisfied, 5 = very satisfied).

Because participants could select any number of items, students who endorsed many items were, by construction, more likely also to endorse self-actualization. The number of items endorsed was therefore compared between the groups and used in a sensitivity analysis in which each value was re-examined alongside the number of the remaining 10 values endorsed.

### Data analysis

Endorsement of the 11 professional values and self-actualization, and mean satisfaction with the major, were compared between the two groups using independent two-sample t-tests with Cohen's d and 95% confidence intervals. Within each group, the association between each value and self-actualization was examined by correlation analysis. Because all items are dichotomous, these statistics are equivalent to a difference between two proportions and to a phi coefficient, and are interpreted as differences in endorsement and as co-endorsement of two binary items rather than as relations between continuous scores.

Binary logistic regression was used to identify the values associated with endorsement of self-actualization; the outcome is dichotomous, so linear regression was not appropriate. All 11 items were entered simultaneously in each group, and odds ratios with 95% confidence intervals, Wald z statistics, the likelihood ratio test, and Nagelkerke R^2^ are reported. Two sensitivity analyses were carried out: the same models were re-estimated as linear probability models, so that the coefficients could also be read as differences in probability,^29^^,^^30^ and each value was re-examined in a model that additionally contained the number of the remaining 10 values endorsed. No a priori model was specified, so all models are exploratory.

Latent groups of students were identified from the 11 dichotomous-valued items using latent class analysis, which is the appropriate specification for categorical indicators, whereas Gaussian mixture models assume continuous indicators.^31^^,^^32^ Models with one to six classes were estimated by maximum likelihood with 40 random starting values, and the number of classes was selected by the Bayesian Information Criterion (BIC), with smaller values indicating better fit.^33^ Classification quality is described by the mean maximum posterior class probability, relative entropy, and the proportion of participants whose maximum posterior probability was below .80, and stability by the adjusted Rand index across 100 bootstrap resamples. Because both samples are small for mixture modeling, the classes are presented as a descriptive summary of recurring patterns of co-endorsement rather than as evidence of distinct subpopulations.

Endorsement of each item was compared across the latent classes using chi-square tests with Cramér's V, and the number of values endorsed was compared using one-way analysis of variance. Because the same 11 items were used both to estimate and to compare the classes, these comparisons are descriptive; only the comparison for self-actualization, which was not used in the estimation, is interpreted substantively.

Because a large number of tests were conducted, the Benjamini–Hochberg procedure was used to control the false discovery rate at .05 within each family of tests: the 13 between-group comparisons and, within each group, the 11 correlations, the 11 logistic coefficients, the 11 adjusted models, and the 12 class comparisons.34 Uncorrected and corrected p values are both reported, and results that did not survive correction are described as not statistically significant. Exact p values are given to three decimal places, with values below .001 reported as p < .001.

All analyses except the latent class analysis were performed with SPSS version 23. The latent class models were estimated in R version 4.4.1 using the poLCA package, and the false discovery rate correction was computed with the p.adjust function.

## Results

### Group differences in values and satisfaction

Endorsement proportions, t-tests, effect sizes, and corrected p-values are presented in Table 2.  Competence was the most frequently endorsed value in both groups (Korean .79, Chinese .62), followed by self-actualization (.60 and .50) and social contribution (.56 and .55). Leadership was the least frequently endorsed item in both groups (.15 and .11), and autonomy (.21 and .16) and creativity (.26 and .18) were also endorsed by relatively few students.

Five of the 13 comparisons remained significant after correction. Korean students endorsed competence, d = 0.38, 95% CI [0.15, 0.62], and collaboration, d = 0.48 [0.24, 0.71], more frequently than Chinese students, and Chinese students endorsed social recognition more frequently, d = −0.35 [−0.59, −0.12]; all three corrected p values were at or below .009. Endorsement of self-actualization did not differ significantly between the groups, d = 0.21 [−0.03, 0.44], nor did the remaining eight value items, all corrected p values above .24. The three significant differences were small, and their confidence intervals indicate considerable uncertainty about the size of the difference. The fifth significant comparison, satisfaction with the major, is reported separately below.

Satisfaction with the major differed markedly between the groups: Korean students reported a mean of 4.36 (SE = .05) and Chinese students a mean of 1.89 (SE = .07), t_(__318)_ = 27.66, p < .001, d = 3.26, 95% CI [2.91, 3.60]. In the Chinese sample, 87.3% of students chose one of the two least favorable options, whereas 82.9% of Korean students chose one of the two most favorable options. Because satisfaction was measured with a single item whose equivalence across the two language versions and the two settings could not be tested, this difference is reported descriptively; it is not interpreted as evidence about either program and is not used in any further analysis.

### Associations with self-actualization

Coefficients quantifying the co-endorsement of each professional value and self-actualization are presented in Table 3.  Among Korean students, seven of the 11 values were associated with endorsement of self-actualization after correction, with coefficients between r = .20 and r = .25; the strongest were social contribution, r = .25, 95% CI [.12, .38], career development, r = .23 [.09, .35], and creativity, r = .22 [.09, .34]. Among Chinese students, five associations survived correction: social contribution, r = .44 [.27, .58], social recognition, r = .31 [.13, .47], career development, r = .29 [.11, .46], leadership, r = .23 [.05, .40], and collaboration, r = .23 [.04, .40]. All coefficients index the extent to which two dichotomous items were selected together and are small to moderate in size; even the largest, social contribution in the Chinese group, corresponds to about 19% shared variance. Results of the logistic regression models are presented in Table 4.

In the Korean group the full model was significant, likelihood-ratio χ²(11) = 41.90, p < .001, Nagelkerke R^2^ = .24, but no individual coefficient remained significant after correction, all corrected p values at or above .214; the largest odds ratios were obtained for creativity (OR = 2.21), social recognition (OR = 2.17), and social contribution (OR = 1.86). The bivariate associations observed in the Korean group therefore did not persist when the 11 values were considered jointly and multiplicity was taken into account.

In the Chinese group the model was also significant, χ²(11) = 39.16, p < .001, Nagelkerke R^2^ = .40, and endorsement of social contribution was the only coefficient to remain significant after correction, OR = 5.42, 95% CI [1.93, 15.25], p = .001. The coefficient for autonomy, OR = 4.55 [1.06, 19.55], p = .042, did not survive correction and had a very wide confidence interval. Because the models were exploratory, these coefficients describe differences in the odds of endorsement within the present samples and should not be read as causal effects.

Two sensitivity analyses supported these results. Linear probability models reproduced the same pattern, and social contribution in the Chinese group was again the only coefficient to survive correction, B = 0.34, 95% CI [0.13, 0.55], p = .002; fitted values fell outside the unit interval for fewer than 5% of participants in either sample. When each value was re-examined together with the number of the remaining values endorsed, that count was itself associated with endorsement of self-actualization in every Korean model, with odds ratios from 1.31 to 1.60 per additional value endorsed, and no single Korean value remained significant after correction, whereas in the Chinese group social contribution was essentially unchanged, OR = 5.57 [2.34, 13.24], p < .001. The Chinese association was therefore not explained by a general tendency to endorse many items, whereas the Korean associations were largely attributable to such a tendency.

**Table 2:** Comparison of endorsement of professional values and satisfaction with major between the two samples

Variable	Korean M (SE)	Chinese M (SE)	t	df	p	p_FDR	Cohen's d [95% CI]
Competence	.79 (.03)	.62 (.05)	3.27	319	= .001	= .005	0.38 [0.15, 0.62]
Diversity	.34 (.03)	.38 (.05)	−0.72	319	= .472	= .614	−0.08 [−0.32, 0.15]
Remuneration	.29 (.03)	.37 (.05)	−1.44	319	= .152	= .282	−0.17 [−0.40, 0.06]
Stability	.38 (.03)	.40 (.05)	−0.36	319	= .717	= .847	−0.04 [−0.27, 0.19]
Social recognition	.31 (.03)	.48 (.05)	−3.01	319	= .003	= .009	−0.35 [−0.59, −0.12]
Leadership	.15 (.02)	.11 (.03)	0.94	319	= .347	= .501	0.11 [−0.12, 0.34]
Collaboration	.42 (.03)	.20 (.04)	4.05	319	< .001	< .001	0.48 [0.24, 0.71]
Social contribution	.56 (.03)	.55 (.05)	0.24	319	= .814	= .882	0.03 [−0.20, 0.26]
Career development	.44 (.03)	.44 (.05)	−0.01	319	= .995	= .995	0.00 [−0.23, 0.23]
Creativity	.26 (.03)	.18 (.04)	1.59	319	= .114	= .247	0.19 [−0.04, 0.42]
Autonomy	.21 (.03)	.16 (.04)	0.97	319	= .335	= .501	0.11 [−0.12, 0.34]
Self-actualization	.60 (.03)	.50 (.05)	1.75	319	= .081	= .210	0.21 [−0.03, 0.44]
Satisfaction with major	4.36 (.05)	1.89 (.07)	27.66	318	< .001	< .001	3.26 [2.91, 3.60]

Note. For the 12 inventory items, the values are endorsement proportions (1 = endorsed, 0 = not endorsed), and the standardized mean difference is interpreted as the standardized difference between two proportions. Satisfaction with the major was rated on a five-point scale. Positive t values indicate higher values in the Korean sample. The degrees of freedom for satisfaction with the major are 318 due to one missing response. p_FDR = p value after Benjamini–Hochberg correction across the 13 comparisons. CI = confidence interval.

**Table 3:** Associations between endorsement of each professional value and endorsement of self-actualization

Professional value	Korean (n = 211) r [95% CI]	p	p_FDR	Chinese (n = 110) r [95% CI]	p	p_FDR
Competence	.21 [.08, .34]	= .002	= .004	.00 [−.19, .19]	= 1.000	= 1.000
Diversity	.10 [−.04, .23]	= .168	= .205	−.11 [−.29, .08]	= .243	= .334
Remuneration	.10 [−.04, .23]	= .150	= .205	.06 [−.13, .24]	= .558	= .682
Stability	.02 [−.12, .15]	= .807	= .807	.04 [−.15, .22]	= .700	= .770
Social recognition	.21 [.08, .34]	= .002	= .004	.31 [.13, .47]	= .001	= .006
Leadership	.06 [−.07, .20]	= .355	= .390	.23 [.05, .40]	= .014	= .037
Collaboration	.20 [.07, .33]	= .003	= .005	.23 [.04, .40]	= .017	= .037
Social contribution	.25 [.12, .38]	< .001	= .002	.44 [.27, .58]	< .001	< .001
Career development	.23 [.09, .35]	< .001	= .004	.29 [.11, .46]	= .002	= .007
Creativity	.22 [.09, .34]	= .001	= .004	.19 [.00, .36]	= .049	= .076
Autonomy	.20 [.07, .33]	= .003	= .005	.20 [.01, .37]	= .040	= .073

Note. Both variables in each pair were dichotomous, so each coefficient is equivalent to a phi coefficient and indexes co-endorsement of two binary items rather than a correlation between continuous measures. Confidence intervals were obtained using Fisher's z-transformation. p_FDR = p value after Benjamini–Hochberg correction across the 11 tests within each sample.

**Table 4:** Logistic regression models of endorsement of self-actualization

Professional value	Korean (n = 211) OR [95% CI]	z	p	p_FDR	Chinese (n = 110) OR [95% CI]	z	p	p_FDR
Competence	1.86 [0.87, 3.99]	1.60	= .111	= .214	1.05 [0.37, 2.99]	0.09	= .929	= .929
Diversity	1.03 [0.50, 2.13]	0.08	= .937	= .937	0.52 [0.19, 1.46]	−1.24	= .215	= .421
Remuneration	1.09 [0.51, 2.31]	0.22	= .824	= .906	0.90 [0.28, 2.92]	−0.17	= .862	= .929
Stability	0.54 [0.25, 1.18]	−1.54	= .123	= .214	0.32 [0.09, 1.15]	−1.75	= .080	= .240
Social recognition	2.17 [0.96, 4.92]	1.86	= .063	= .214	3.30 [0.84, 12.97]	1.71	= .087	= .240
Leadership	0.80 [0.31, 2.12]	−0.44	= .659	= .805	1.99 [0.28, 13.93]	0.69	= .489	= .686
Collaboration	1.76 [0.87, 3.58]	1.56	= .118	= .214	2.43 [0.57, 10.35]	1.20	= .229	= .421
Social contribution	1.86 [0.98, 3.53]	1.90	= .057	= .214	5.42 [1.93, 15.25]	3.20	= .001	= .015
Career development	1.59 [0.81, 3.11]	1.35	= .175	= .241	1.53 [0.45, 5.19]	0.68	= .499	= .686
Creativity	2.21 [1.01, 4.82]	1.99	= .046	= .214	0.74 [0.16, 3.31]	−0.40	= .690	= .843
Autonomy	1.97 [0.81, 4.82]	1.49	= .136	= .214	4.55 [1.06, 19.55]	2.04	= .042	= .229

Note. Binary logistic regression with endorsement of self-actualization as the dependent variable and the 11 professional values entered simultaneously; separate models were fitted in each sample. OR = odds ratio; a value above 1 indicates higher odds of endorsing self-actualization among students who endorsed the value in question, holding the remaining 10 values constant. p_FDR = p value after Benjamini–Hochberg correction across the 11 coefficients within each sample. Linear probability models fitted to the same data are reported in the text as a sensitivity analysis. Korean model: χ²(11) = 41.90, p < .001, Nagelkerke R^2^ = .24. Chinese model: χ²(11) = 39.16, p < .001, Nagelkerke R^2^ = .40.

### Latent value classes

The BIC favored a two-class solution in the Korean group and a three-class solution in the Chinese group, although in the Chinese group the two- and three-class values differed by only 3.3, so the number of classes is weakly determined. Class-specific endorsement proportions are reported in Table 5. Classification quality was acceptable but not high. In the Korean solution the mean maximum posterior probability was .93, relative entropy was .75, and 13.3% of participants had a maximum posterior probability below .80; the mean adjusted Rand index across 100 bootstrap resamples was 0.70. The corresponding values in the Chinese solution were .94, .86, 10.9%, and 0.51. The Chinese solution is therefore appreciably less stable than the Korean one, and both are treated as descriptive.

In the Korean group, Class 1 (n = 144, 68.2%) endorsed few values, a mean of 2.81 (SD = 1.46) of the 11 items, most often competence (.69) and social contribution (.47) and rarely leadership (.06), autonomy (.13), or social recognition (.13). Class 2 (n = 67, 31.8%) endorsed a mean of 7.03 (SD = 1.60) items, with competence endorsed by all members and no item below .34. The two classes differed markedly in the number of values endorsed, F(1, 209) = 359.40, p < .001.

In the Chinese group, Class 1 (n = 53, 48.2%) endorsed a mean of 4.58 (SD = 1.29) items and was characterized by social recognition (.81), social contribution (.74), and career development (.60). Class 2 (n = 47, 42.7%) endorsed a mean of 1.91 (SD = 1.19) items, with competence (.57) the only item endorsed by more than half and no endorsement of social recognition or leadership. Class 3 (n = 10, 9.1%) endorsed a mean of 9.50 (SD = 1.08) items, that is, nearly all of them; with only ten members, its profile is estimated imprecisely. The three classes differed in the number of values endorsed, F(2, 107) = 171.85, p < .001.

Comparisons of the 11 estimation indicators across classes were significant for most items in both groups, but these comparisons are circular because the same items defined the classes; they are reported in Table 6 for descriptive purposes only.  The one non-circular comparison concerns self-actualization, which was not used to estimate the models. Endorsement was .51 in Korean Class 1 and .81 in Class 2, χ²(1) = 17.06, p < .001, Cramér's V = .28, and .57, .34, and .90 in Chinese Classes 1 to 3, χ²(2) = 12.11, p = .002, Cramér's V = .33. In both groups endorsement of self-actualization was highest in the class that endorsed most items, so this difference cannot be separated from the difference between classes in breadth of endorsement.

## Discussion

This study compared the professional values endorsed by medical students at one Korean and one Chinese institution and examined their association with endorsement of self-actualization. Four findings concerning professional values withstood correction for multiplicity: Korean students endorsed competence and collaboration more frequently, Chinese students endorsed social recognition more frequently, and endorsement of social contribution was associated with endorsement of self-actualization in both groups, remaining significant in the Chinese multivariable model. Satisfaction with the major was also far higher in the Korean group, but this single-item comparison is reported descriptively and is not interpreted. All other differences and associations were either small or did not survive correction, and the following discussion is confined to these findings.

### Values endorsed in both groups

The high endorsement of competence and social contribution in both groups is consistent with previous reports that medical students prioritize clinical capability and service to society among the attributes they associate with the profession.^35^ Comparable patterns have been described among Korean physicians, for whom competence and public responsibility rank highly among professional attributes,^35^ and in Chinese-language research on medical professionalism, in which service to patients and society is a recurring core theme.^36^^,^^37^ The present results extend these single-setting findings by showing that the same two items are the most frequently endorsed in two different institutional settings.

Leadership, creativity, and autonomy were endorsed by relatively few students in both groups. This is consistent with descriptions of medical curricula in which standardized knowledge and skill acquisition and supervised clinical training predominate,^38^^,^^39^ and with reports that leadership is seldom made explicit as a learning objective in undergraduate medical programs. The present data, however, contain no measure of curricular content, teaching method, assessment design, or institutional climate. The correspondence with that literature is therefore noted as consistent with previous descriptive accounts and not as evidence that curricular or institutional features account for the observed endorsement rates. Explanations of this kind require designs in which such features are measured.

### Differences between the two groups

The differences in competence, collaboration, and social recognition were small and their confidence intervals were wide. Because each country was represented by a single institution, and because the samples were not matched on sex or academic year, these differences cannot be attributed to national culture. They may equally reflect differences between the two institutions in admissions, curriculum, year-level composition, teaching format, or the setting in which the questionnaire was completed. The large difference in satisfaction with the major is reported descriptively for the same reason: it was measured by a single item in two language versions whose equivalence was not established, and a difference of that magnitude cannot be interpreted without evidence that the item functioned equivalently in the two settings.

**Table 5:** Endorsement proportions for each professional value by latent class

Professional value	Korea Class 1 (n = 144)	Korea Class 2 (n = 67)	China Class 1 (n = 53)	China Class 2 (n = 47)	China Class 3 (n = 10)
Competence	.69	1.00	.58	.57	1.00
Diversity	.22	.60	.32	.38	.70
Remuneration	.19	.51	.42	.19	1.00
Stability	.20	.76	.58	.09	.90
Social recognition	.13	.70	.81	.00	1.00
Leadership	.06	.34	.08	.00	.80
Collaboration	.25	.79	.19	.09	.80
Social contribution	.47	.76	.74	.26	.90
Career development	.26	.82	.60	.13	1.00
Creativity	.21	.37	.21	.02	.80
Autonomy	.13	.37	.06	.19	.60
Self-actualization	.51	.81	.57	.34	.90
Number of the 11 values endorsed, M (SD)	2.81 (1.46)	7.03 (1.60)	4.58 (1.29)	1.91 (1.19)	9.50 (1.08)
Proportion of the sample	.68	.32	.48	.43	.09

Note. Values are within-profile endorsement proportions obtained from latent class analysis of the 11 dichotomous value items, conducted separately in each sample. The number of classes was selected by the Bayesian Information Criterion, which favored two classes in the Korean sample and three in the Chinese sample. Self-actualization and the number of values endorsed were not used to estimate the models.

**Table 6:** Differences in endorsement across the latent classes

Variable	Korea χ²(1)	p	p_FDR	Cramér's V	China χ²(2)	p	p_FDR	Cramér's V
Competence	26.61	< .001	< .001	.36	6.81	= .033	= .036	.25
Diversity	28.57	< .001	< .001	.37	5.13	= .077	= .077	.22
Remuneration	21.59	< .001	< .001	.32	23.84	< .001	< .001	.47
Stability	60.87	< .001	< .001	.54	37.39	< .001	< .001	.58
Social recognition	69.00	< .001	< .001	.57	77.50	< .001	< .001	.84
Leadership	30.20	< .001	< .001	.38	55.49	< .001	< .001	.71
Collaboration	54.88	< .001	< .001	.51	26.42	< .001	< .001	.49
Social contribution	16.24	< .001	< .001	.28	28.78	< .001	< .001	.51
Career development	59.14	< .001	< .001	.53	37.17	< .001	< .001	.58
Creativity	6.44	= .011	= .011	.17	34.07	< .001	< .001	.56
Autonomy	16.12	< .001	< .001	.28	18.62	< .001	< .001	.41
Self-actualization	17.06	< .001	< .001	.28	12.11	= .002	= .003	.33

Note. Chi-square tests of association between latent class membership and endorsement of each item. The comparisons for the 11 professional values are circular because the same items were used to estimate the latent class models; they are reported for descriptive purposes only. Self-actualization was not used in the estimation and is the only non-circular comparison. p_FDR = p-value after Benjamini–Hochberg correction across the 12 comparisons within each sample.

Previous cross-national work has reported that the meaning and expression of medical professionalism vary across settings and that professionalism frameworks developed in one context cannot be assumed to transfer unchanged to another.^14^ The present findings are consistent with the more limited claim that student groups in different settings endorse somewhat different values; they do not identify the source of that variation. Although SDT and Hofstede's dimensions were used to frame the study, neither need satisfaction nor cultural orientation was measured, so the data cannot adjudicate among motivational, curricular, institutional, and compositional accounts of the observed differences.

### Association between social contribution and self-actualization

Endorsement of social contribution was associated with endorsement of self-actualization in both groups, and in the Chinese group, it was the only multivariable coefficient to remain significant after FDR correction and after adjustment for the number of other values endorsed. This is consistent with accounts of medicine as a vocation in which service to others is central to professional fulfillment,^40^ and with research reporting that prosocial goals are associated with well-being and engagement among students.^41^^,^^42^ The association is cross-sectional and between two dichotomous items, and the direction of the relationship cannot be established: students who see their future work as socially valuable may be more likely to describe it as self-actualizing, or students who seek self-actualization may be more likely to endorse socially oriented values, or both endorsements may reflect a shared tendency to endorse ideal professional attributes.

Two features of the measurement limit interpretation further. Self-actualization was assessed by a single dichotomous item within the same checklist as the value items, so shared method variance is likely and the observed associations may be inflated.^43^ In addition, because the number of items endorsed was unrestricted, students with a general tendency to endorse many items showed positive associations across pairs of items. The present analyses indicate that this tendency accounts for a substantial part of the pattern: in the Korean group, the number of other values endorsed was associated with endorsement of self-actualization in every adjusted model, and no individual value remained significant once it was controlled, whereas in the Chinese group, the association with social contribution was essentially unchanged by this adjustment. The Chinese finding for social contribution is therefore the more robust of the two, while the Korean bivariate associations are best interpreted as reflecting breadth of endorsement rather than the specific content of individual values. For these reasons, no claim is made about the level, degree, or development of self-actualization; the analyses concern only whether students selected self-actualization as an important professional value.

### Heterogeneity within groups

Two latent classes were identified in the Korean group and three in the Chinese group, and endorsement of self-actualization differed across classes in both. The classes differed above all in how many values students endorsed, from a mean of 2.81 to 7.03 items in the Korean group and from 1.91 to 9.50 items in the Chinese group, so they are best described as reflecting breadth of endorsement, together with a contrast in the Chinese sample between students oriented toward social recognition and contribution and students endorsing few values of any kind. Comparisons of the estimation indicators across classes are circular by construction; the difference in endorsement of self-actualization is not, but because it follows the same breadth gradient it cannot be interpreted as evidence that particular value patterns accompany self-actualization. Given the modest bootstrap stability of both solutions and the very small third Chinese class, the classes are treated as a descriptive summary rather than as evidence of distinct subpopulations.

The practical observation supported by these results is that students within the same institution and year level did not endorse a single common value pattern. This within-group variation is consistent with research reporting that medical students' career preferences and orientations differ systematically within cohorts,^44^^,^^45^ and it suggests that career guidance addressed to an assumed common set of professional priorities may not fit all students in a cohort. This is offered as a hypothesis for evaluation in studies designed for that purpose, not as a demonstrated effect of any educational intervention.

### Implications for medical education

Three implications follow directly from the results. First, because the value patterns within each group were heterogeneous, career advising and professional-identity teaching may benefit from eliciting students' individual value priorities rather than assuming a shared set. Simple instruments of the kind used here can support such discussions, although they are not diagnostic. Second, because endorsement of social contribution was a correlate of self-actualization common to both groups and was robust to adjustment in the Chinese sample, curricular activities that make the social value of clinical work explicit, such as structured reflection on the profession's societal role, may be relevant to students in both settings. Third, for international exchange programs, the present findings indicate that students arriving from different institutions may prioritize different professional attributes; program designers may therefore find it useful to establish participants' value priorities directly rather than inferring them from national origin, which this study cannot support as a basis for such inference. These implications are advanced tentatively, since the study measured neither educational processes nor outcomes.

### Limitations

This study has several limitations. First, each country was represented by a single institution, and participants were recruited via convenience sampling, so the findings cannot be generalized to medical students in South Korea or China, and observed differences cannot be attributed to country or culture. Second, the groups were not matched on year of training: all Chinese participants were at the stage corresponding to the Korean second year, whereas 44.1% of the Korean participants were in their third year, and the correspondence between year levels in the two curricula is approximate rather than formally established. Age was not collected, and the groups were not matched on sex. Compositional differences may therefore contribute to the between-group differences reported. Third, the instrument was developed for Korean secondary and vocational education and has not been psychometrically evaluated among Chinese medical students; measurement invariance was not tested, so equivalence of item meaning across the two versions is assumed rather than demonstrated,^28^ and the between-group comparisons rest on that assumption. Fourth, all value constructs were measured by single dichotomous items with unrestricted multiple selection, which precludes assessment of internal consistency, provides no information about the strength of endorsement, and makes the results sensitive to individual differences in the number of items selected; the sensitivity analyses reported above show that this sensitivity is material in the Korean sample. Fifth, self-actualization was measured within the same checklist as the predictors, so common method variance may have inflated the associations.^43^ Sixth, satisfaction with the major was measured by a single unvalidated item, and the very large between-group difference in this variable cannot be interpreted: differential interpretation of the response categories, differences in the setting in which the questionnaire was administered, and translation of the response labels are all plausible contributors, and none can be examined with these data. This comparison is therefore reported descriptively only. Seventh, the latent class solutions were estimated in small samples; the three-class Chinese solution was only weakly favored over the two-class solution and included a class of 10 students; bootstrap stability was moderate in the Korean sample and modest in the Chinese sample; the classes are therefore exploratory. Eighth, the design was cross-sectional, so no statement can be made about development during training or about the direction of any association, and all analyses were exploratory rather than confirmatory. Ninth, the data were collected at a single time point in each institution, and no information was available on non-respondents, so the extent of any selection bias cannot be estimated.

Future research should sample multiple institutions in each country so that institutional and national levels can be separated, establish measurement invariance before making cross-national comparisons, use multi-item graded measures of professional values and self-actualization that are not vulnerable to differences in the number of items selected, adopt longitudinal designs to examine change across training, and complement survey data with interviews or focus groups to clarify how students themselves understand these values.

## Conclusions

This study examined the professional values endorsed by medical students at one Korean and one Chinese institution and their association with endorsement of self-actualization. Competence, self-actualization and social contribution were the most frequently endorsed items in both groups; Korean students endorsed competence and collaboration more frequently and Chinese students endorsed social recognition more frequently, with small effects; and endorsement of social contribution was the value most consistently associated with endorsement of self-actualization, remaining significant in the Chinese sample after correction for multiplicity and after adjustment for the number of other values endorsed, while the Korean associations were largely attributable to differences in how many values students endorsed. Latent class analysis indicated heterogeneity within both groups, consisting mainly of differences in the breadth of endorsement. Because each country was represented by a single institution and the instrument has not been validated for cross-national comparison, these results describe two student groups and do not establish cultural differences. For medical education, they suggest that career guidance and professional-identity teaching may benefit from attending to the diversity of value priorities within a cohort and from making the social contribution of clinical work explicit. Multi-institutional studies using invariance-tested, multi-item instruments and longitudinal designs are needed to determine whether these patterns hold more widely and how they develop during training.

### Conflict of Interest

The authors declare no conflicts of interest.
